# The Association Between the Nursing Practice Environment and Culturally Competent Behaviors Among Surgical Nurses in Riyadh, Saudi Arabia: A Multicenter Cross-Sectional Study

**DOI:** 10.3390/healthcare14183017

**Published:** 2026-09-15

**Authors:** Alwah Mohammed Alkathiri, Manal Fehade Alharbi

**Affiliations:** 1 Medical Surgical Nursing, College of Nursing, King Saud University, Riyadh 14242, Saudi Arabia; 2 Maternal & Child Health Department, College of Nursing, King Saud University, Riyadh 14242, Saudi Arabia; maalwahbi@ksu.edu.sa

**Keywords:** cultural competence, nursing work environment, practice environment, Saudi Arabia, surgical nursing

## Abstract

**Highlights:**

**What are the main findings?**

- A moderate positive association was observed between the nursing practice environment and culturally competent behavior scores (ρ = 0.303); the association remained statistically significant after adjustment (B = 1.037, *p* < 0.001).
- The staffing and resource adequacy domain received the lowest rating among the nursing practice environment domains.

**What are the implications of the main findings?**

- Cultural competence initiatives should be supported by effective nursing leadership, adequate staffing and resources, interprofessional collaboration, and culturally appropriate communication support.
- Longitudinal and intervention studies are needed to clarify causality and determine whether improvements in the practice environment enhance culturally competent care and patient outcomes.

**Abstract:**

Background/Objectives: Culturally competent nursing care is essential in multicultural settings, but organizational conditions may influence its delivery. This study examined the association between the nursing practice environment and culturally competent behaviors among surgical nurses in Riyadh, Saudi Arabia, and whether this association remained significant after adjustment. Methods: This multicenter cross-sectional study included 422 surgical nurses recruited by convenience sampling from January to March 2026. Participants completed the 31-item Practice Environment Scale of the Nursing Work Index (PES-NWI) and the 14-item Cultural Competence Behaviors (CCB) subscale of the Cultural Competence Assessment. Analyses included descriptive statistics, Cronbach’s alpha, Spearman’s rank correlation, and an adjusted general linear model with heteroscedasticity-consistent type 3 (HC3)-robust standard errors. Results: The mean PES-NWI score was 3.04 ± 0.46; nursing foundations for quality of care had the highest domain score (3.16 ± 0.43), whereas staffing and resource adequacy had the lowest (2.87 ± 0.63). Among the 404 participants with valid CCB scores, the mean CCB score was 4.48 ± 1.53. The PES-NWI composite score showed a moderate positive association with CCB (ρ = 0.303, *p* < 0.001). The adjusted model was statistically significant (F(14, 389) = 8.28, *p* < 0.001; R^2^ = 0.230). Adding the PES-NWI composite score explained an additional 9.1% of the variance in CCB (ΔR^2^ = 0.091, *p* < 0.001); the adjusted association remained positive (B = 1.037, HC3-robust standard error = 0.172, 95% confidence interval = 0.699–1.375, *p* < 0.001). Conclusions: The nursing practice environment had a moderate positive unadjusted association with CCB, and this association remained statistically significant after adjustment. Longitudinal studies are needed to clarify the temporal direction of this relationship.

## 1. Introduction

Cultural competence is important for respectful and patient-centered care in increasingly diverse healthcare systems [1]. This is particularly relevant in Saudi Arabia, where a multinational nursing workforce provides care within a culturally and linguistically diverse healthcare environment [2,3]. Patients’ cultural values, religious beliefs, language, family roles, and social expectations may influence communication, healthcare decisions, and responses to care [1,4]. In this context, culturally competent nursing practice extends beyond awareness of cultural differences and involves translating cultural awareness and sensitivity into behaviors that respond appropriately to individual patient needs [2,3].

Cultural considerations are especially relevant in surgical settings, where nurses contribute to preoperative preparation, postoperative monitoring, pain management, privacy protection, family communication, and discharge education. Cultural and religious expectations may influence modesty, gender preferences, family involvement, prayer, and dietary practices [4]. In Saudi healthcare settings, linguistic and cultural differences may complicate communication between patients and healthcare professionals [5,6]. Informed consent may present additional challenges in time-sensitive surgical situations, particularly when decision-making capacity is limited or when little time is available [7]. Cultural background may also influence the perception, expression, and management of pain [8]. Together, these factors may complicate nurses’ efforts to individualize care in surgical settings.

Research on cultural competence has commonly emphasized individual-level components, including cultural awareness, knowledge, sensitivity, and related competencies [9]. However, individual competence alone may not fully explain the delivery of culturally competent care in practice. Organizational conditions can facilitate or constrain its implementation. Staffing challenges, insufficient culturally appropriate policies, and limited training have been identified as barriers to culturally competent care [10], while nurse leadership may help create practice environments that support nurses in applying culturally appropriate care practices [11].

The nursing practice environment comprises the organizational characteristics that support or hinder professional nursing practice. The Practice Environment Scale of the Nursing Work Index (PES-NWI) assesses five dimensions of the professional nursing practice environment [12,13]. Favorable practice environments have been associated with improved nurse, patient, and organizational outcomes [14]. However, evidence examining the relationship between the nursing practice environment and culturally competent behaviors remains limited. A recent study in Portuguese multicultural healthcare units found that cultural competence and the nursing work environment were associated with culturally congruent care [15]. Comparable evidence among surgical nurses in Saudi Arabia remains scarce.

This study was guided by Schim and colleagues’ Three-Dimensional Model of Cultural Congruence and informed by Donabedian’s Structure–Process–Outcome framework. Schim’s model conceptualizes cultural competence through cultural diversity, cultural awareness, cultural sensitivity, and culturally competent behaviors. In the present study, culturally competent behaviors refer to the actions used by nurses to respond appropriately to cultural differences during clinical care [16,17] and, specifically, to the behavioral component measured by the Cultural Competence Assessment (CCA) [18]; the term is not used interchangeably with the broader concept of culturally congruent care. In this study, Donabedian’s framework was used to position the nursing practice environment as an organizational structure and culturally competent behaviors as a nursing care process. The PES-NWI was selected to operationalize the structural component because the study focused specifically on the nursing practice environment, and its five domains capture key organizational conditions within which professional nursing care is delivered [12,13]. These domains therefore align conceptually with Donabedian’s structural component [19]. The five domains include nurse participation in hospital affairs; nursing foundations for quality of care; nurse-manager ability, leadership, and support of nurses; staffing and resource adequacy; and collegial nurse–physician relations. Although other structural characteristics, such as workload and interpreter availability, may also influence culturally competent care, this study did not assess them as separate variables. Accordingly, this operationalization represents a defined component of organizational structure rather than an exhaustive assessment of all structural factors. Supportive leadership, adequate staffing and resources, nurse participation, foundations for quality of care, and effective nurse–physician relationships may strengthen nurses’ capacity to translate cultural awareness and sensitivity into practice. Because patient outcomes were not measured, the study focused on the association between organizational structure and the nursing care process rather than the complete Structure–Process–Outcome pathway [19].

Despite the recognized importance of cultural competence and the nursing practice environment, the relationship between them has been insufficiently studied. Existing work has largely emphasized individual-level cultural competence or broader nursing populations, leaving two related gaps: whether the organizational nursing practice environment is associated with a behavioral measure of cultural competence and whether this relationship is evident in the time-sensitive context of surgical nursing in Saudi Arabia. To the best of our knowledge, no previous study has specifically examined this association among surgical nurses in Saudi Arabia. The present study therefore addresses both an organizational-level and a specialty-specific gap by linking the PES-NWI to reported culturally competent behaviors while accounting for study site and selected demographic and professional characteristics.

This study aimed to examine the association between the nursing practice environment and culturally competent behaviors among surgical nurses in Riyadh, Saudi Arabia, and to determine whether this association remained significant after adjustment for study site and selected demographic and professional characteristics.

The following hypotheses were tested:

**H1.**  *A more favorable nursing practice environment would be positively associated with higher culturally competent behavior scores.*

**H2.**  *The nursing practice environment would remain significantly associated with culturally competent behaviors after adjustment for study site and selected demographic and professional characteristics.*

## 2. Materials and Methods

### 2.1. Study Design and Setting

A multicenter cross-sectional study was conducted among registered nurses working in surgical units across three large tertiary healthcare institutions in Riyadh, Saudi Arabia, representing different organizational settings. All three participating institutions were government-operated tertiary healthcare institutions, one of which also had a formal teaching role. To preserve institutional anonymity, the participating institutions are referred to as Site A, Site B, and Site C throughout the manuscript. The sites were purposively selected to capture variation in nursing practice environments and patient populations. One large tertiary referral center was selected from each of two Riyadh health clusters, and a third Magnet-recognized tertiary referral institution was included to broaden organizational variation. The participating institutions receive referrals from different regions of Saudi Arabia, providing surgical nurses with exposure to patients from diverse regional, cultural, and social backgrounds. Data were collected from January to March 2026 using an online self-administered questionnaire. The study was reported in accordance with the Strengthening the Reporting of Observational Studies in Epidemiology (STROBE) guidelines for cross-sectional studies.

### 2.2. Participants

Inclusion criteria: Registered nurses were eligible if they worked in inpatient or day-care surgical units, were directly involved in bedside nursing care, had at least one year of experience in their current surgical setting, and were able to read and understand English.

Exclusion criteria: Nurses were excluded if they were not involved in direct patient care.

### 2.3. Sample Size and Sampling Technique

Convenience sampling was used to recruit eligible surgical nurses who were available and willing to participate during the study period.

The minimum sample size was calculated a priori using G*Power version 3.1.9.7 (Heinrich Heine University Düsseldorf, Düsseldorf, Germany) for multiple linear regression with a fixed model and R^2^ increase. Assuming a small effect size of f^2^ = 0.02, one tested predictor, 19 total predictor degrees of freedom, a significance level of 0.05, and 80% power, the minimum required sample was 395 participants. The a priori calculation used a conservative pre-analysis assumption of 19 predictor degrees of freedom; the finalized adjusted model used fewer degrees of freedom after consolidation of prespecified categories.

Across the three participating institutions, 1030 surgical nurses met the eligibility criteria and were invited to participate. A total of 422 questionnaires were received, yielding a response rate of 41.0%. Of these, 404 contained sufficient valid responses to calculate the culturally competent behavior score and were included in the correlation and adjusted analyses.

### 2.4. Data Collection Instrument

Data were collected in English using an online self-administered questionnaire accessed through an electronic survey link or quick response (QR) code. No translated versions were used because the ability to read and understand English was an eligibility criterion. Accordingly, no translation, back-translation, or language-specific cultural adaptation procedures were undertaken. The questionnaire contained three sections.

The first section collected demographic and professional information, including age, gender, study site, nationality, religion, educational level, and nursing experience. Religion was recorded using two predefined response categories, Muslim and Others; specific religious affiliations within the Others category were not collected.

The second section assessed the nursing practice environment using the 31-item PES-NWI [12,13]. The scale contains five domains: nurse participation in hospital affairs; nursing foundations for quality of care; nurse-manager ability, leadership and support of nurses; staffing and resource adequacy; and collegial nurse–physician relations. Responses ranged from 1, “strongly agree,” to 4, “strongly disagree.” Items were reverse-coded so that higher scores indicated a more favorable nursing practice environment. Domain scores were calculated as the means of their respective items, and the overall PES-NWI composite score was calculated as the mean of the five domain scores.

The third section assessed culturally competent behaviors using the Cultural Competence Behaviors (CCB) subscale of the CCA. The original CCA was developed as a 25-item instrument [17]; a subsequent 25-item version comprises an 11-item Cultural Awareness and Sensitivity subscale and a 14-item CCB subscale [18]. Because this study focused specifically on culturally competent behaviors, only the 14-item CCB subscale was administered. CCB items were rated on a 7-point frequency scale from 1, “never,” to 7, “always,” with higher scores indicating more frequent culturally competent behaviors; “not sure” was a non-scored response option. The CCB score was calculated as the mean of valid item responses. For this study, a CCB score was calculated only when at least 11 of the 14 items had valid responses; this was a study-specific completeness criterion rather than a scoring requirement attributed to the instrument developers.

Written permission to use the PES-NWI and CCA instruments was obtained from the respective authors before data collection.

### 2.5. Study Variables

The primary independent variable was the PES-NWI composite score, representing the overall nursing practice environment, and the dependent variable was the 14-item CCB subscale of the CCA. The five established PES-NWI domains were also examined descriptively and in secondary domain-level analyses. The CCB subscale was analyzed as a single composite outcome because the study focused specifically on culturally competent behaviors rather than the broader CCA construct. H1 was evaluated using Spearman’s rank correlation between the PES-NWI composite score and CCB, whereas H2 was evaluated using the adjusted general linear model.

The adjusted analysis included study site, age group, gender, nationality, religion, educational level, and nursing experience. These variables were entered as categorical factors, whereas the PES-NWI composite score was entered as a continuous predictor. These adjustment variables were selected because of their potential relevance to culturally competent behaviors and the nursing practice environment, based on the study framework and previous literature.

### 2.6. Statistical Analysis

Data were analyzed using the Statistical Package for the Social Sciences (SPSS), IBM SPSS Statistics for Windows, Version 29.0 (IBM Corp., Armonk, NY, USA). Frequencies and percentages were used to summarize categorical variables; means and standard deviations were used for scale-derived variables. Internal consistency was assessed using Cronbach’s alpha.

For CCB scoring, “not sure” responses were not scored and were excluded from the participant-level mean, which was calculated from valid item responses scored from 1 to 7. A study-specific completeness criterion required at least 11 of the 14 CCB items to have valid responses for a participant-level CCB score to be calculated. Consequently, 404 of the 422 participants were included in analyses involving CCB. Because the PES-NWI composite and CCB scores departed from normality, Spearman’s rank correlation coefficient was used to examine their unadjusted association. Secondary Spearman rank correlations were also calculated between each of the five PES-NWI domain scores and CCB.

An adjusted general linear model was used to assess the association between the PES-NWI composite score and CCB, adjusting for study site, age group, gender, nationality, religion, educational level, and nursing experience. The PES-NWI composite score was entered as a continuous predictor, whereas the adjustment variables were entered as categorical factors. Heteroscedasticity-consistent type 3 (HC3)-robust standard errors and 95% confidence intervals were reported. Model fit was summarized using R^2^ and adjusted R^2^. All tests were two-sided, and statistical significance was set at *p* < 0.05. Multicollinearity was assessed using tolerance and variance inflation factor (VIF) statistics. The final model showed no evidence of severe multicollinearity, with VIF values ranging from 1.10 to 4.14 and tolerance values ranging from 0.24 to 0.91. Linearity of the association between the PES-NWI composite score and CCB was examined by adding a quadratic PES-NWI term to the adjusted model. A sensitivity analysis was used to determine whether allowing for curvature materially changed model fit or the substantive interpretation of the association. To examine whether participants with and without calculable CCB scores differed in their demographic and professional characteristics, participants with calculable CCB scores (*n* = 404) were compared with those for whom a CCB score could not be calculated (*n* = 18). Pearson’s chi-square tests were used when assumptions were met, whereas exact tests were used when expected cell counts were sparse. The bivariate analysis presents unadjusted rank-based associations, whereas the adjusted analysis presents the association between the PES-NWI composite score and CCB after adjustment for the prespecified covariates. The two analyses address different questions, and their coefficients are not directly comparable. As an additional sensitivity analysis, the primary bivariate and adjusted analyses were repeated among participants with complete responses to all 14 CCB items (complete-case sample, *n* = 345) to assess whether the findings were sensitive to the study-specific ≥ 11-item completeness criterion.

For secondary comparisons of CCB scores across demographic and professional characteristics, Mann–Whitney U tests were used for variables with two groups and Kruskal–Wallis tests for variables with three or more groups. For consistency with the final adjusted model, age was analyzed in three categories (26–35 years, 36–45 years, and ≤25 or ≥46 years), and nationality was consolidated into four categories (Indian, Filipino, Saudi, and other nationalities). Regression diagnostics included inspection of standardized and studentized residuals, residual-versus-predicted plots, residual normality, Cook’s distance, and leverage values to assess model assumptions and potentially influential observations.

### 2.7. Ethical Considerations

The study was conducted in accordance with the Declaration of Helsinki and received ethical approval from the relevant institutional review boards responsible for the participating healthcare institutions in Riyadh, Saudi Arabia. Administrative permission was obtained from each participating institution before data collection. Data collection at each institution began only after the corresponding ethical approval had been obtained. Participation was voluntary, electronic informed consent was obtained before questionnaire completion, and no personal identifiers were collected.

## 3. Results

### 3.1. Sample Characteristics

A total of 422 surgical nurses participated in the study. Most participants were female (*n* = 377, 89.3%). The largest age groups were 26–35 years (*n* = 173, 41.0%) and 36–45 years (*n* = 160, 37.9%). Indian nurses formed the largest nationality group (*n* = 168, 39.8%), followed by Filipino nurses (*n* = 124, 29.4%) and Saudi nurses (*n* = 70, 16.6%). Regarding the predefined religion categories, 123 participants (29.1%) were Muslim, whereas 299 (70.9%) were classified in the Others category. Most participants held a bachelor’s degree or higher (*n* = 354, 83.9%), and nearly half had ≥10 years of nursing experience (*n* = 202, 47.9%) (Table 1).

Of the 422 participants, 404 (95.7%) had calculable CCB scores. The remaining 18 participants completed the questionnaire but had fewer than 11 scorable CCB item responses because “not sure” responses were not scored; therefore, they did not meet the study-specific completeness criterion. Comparisons between participants with and without calculable CCB scores showed no statistically significant differences in gender, age group, religion, educational level, or nursing experience. Differences were observed by study site (χ^2^(2) = 26.97, *p* < 0.001) and nationality (exact *p* = 0.041).

### 3.2. Reliability of the Study Measures

Both instruments demonstrated high internal consistency. Cronbach’s alpha was 0.964 for the overall PES-NWI and ranged from 0.802 to 0.926 across its five domains. For the CCB scale, Cronbach’s alpha was 0.955, based on the 345 participants who provided complete responses to all 14 items (Table 2).

### 3.3. Nursing Practice Environment and Culturally Competent Behaviors

The mean PES-NWI composite score was 3.04 ± 0.46 on a 1–4 scale, indicating that the nursing practice environment was perceived favorably overall. Among the five domains, nursing foundations for quality of care had the highest mean score (3.16 ± 0.43), followed by collegial nurse–physician relations (3.11 ± 0.50). Staffing and resource adequacy had the lowest mean score (2.87 ± 0.63), indicating that it was the least favorably perceived domain, although its mean remained above the scale midpoint. The mean CCB score was 4.48 ± 1.53 on a 1–7 scale and was above the scale midpoint (Table 3).

### 3.4. Bivariate Association with Culturally Competent Behaviors

Because the PES-NWI composite and CCB scores were not normally distributed, Spearman’s rank correlation was used to examine the unadjusted association. A statistically significant positive association was found between the nursing practice environment and culturally competent behaviors (ρ = 0.303, *p* < 0.001, N = 404). Nurses who reported a more favorable practice environment tended to report higher culturally competent behavior scores. The association was moderate in magnitude and supported the first study hypothesis (Table 4). Secondary domain-level analyses showed statistically significant positive associations between CCB and all five PES-NWI domains. The strongest association was observed for nurse participation in hospital affairs (ρ = 0.332), followed by nursing foundations for quality of care (ρ = 0.309), staffing and resource adequacy (ρ = 0.275), nurse-manager ability, leadership and support of nurses (ρ = 0.265), and collegial nurse–physician relations (ρ = 0.249); all *p* < 0.001. Overall, these domain-level associations were modest in magnitude.

### 3.5. Adjusted General Linear Model

The overall adjusted model was statistically significant—F(14, 389) = 8.28, *p* < 0.001—and explained 23.0% of the variance in culturally competent behavior scores (R^2^ = 0.230; adjusted R^2^ = 0.202). In a hierarchical comparison, the covariates alone explained 13.9% of the variance in CCB scores (R^2^ = 0.139). Adding the PES-NWI composite score increased the total explained variance to 23.0%, an additional 9.1 percentage points (ΔR^2^ = 0.091, *p* < 0.001). After adjustment for study site and selected demographic and professional characteristics, the nursing practice environment remained positively and significantly associated with culturally competent behaviors. Each one-point increase in the PES-NWI composite score was associated with an average 1.04-point increase in the culturally competent behavior score (B = 1.037, HC3-robust standard error = 0.172, 95% confidence interval = 0.699–1.375, *p* < 0.001). This finding supported the second study hypothesis (Table 5). The analyses in Table 4 and Table 5 address different questions. Table 4 reports the unadjusted monotonic association between PES-NWI and CCB, whereas Table 5 estimates the adjusted association while accounting for study site and demographic and professional covariates. Accordingly, Spearman’s ρ and the R^2^ of the full adjusted model are not directly comparable. The identity R^2^ = r^2^ applies only to simple linear regression with a single predictor when r is the Pearson correlation coefficient, which is not the analysis presented here.

Abbreviations: PES-NWI, Practice Environment Scale of the Nursing Work Index; HC3, heteroscedasticity-consistent type 3; SE, standard error; CI, confidence interval. Dependent variable: Cultural Competence Behaviors. N = 404. The model was adjusted for age group, gender, study site, nationality, religion, educational level, and nursing experience. R^2^ = 0.230; adjusted R^2^ = 0.202; F(14, 389) = 8.28, *p* < 0.001. HC3-robust standard errors were reported. R^2^ refers to the variance explained by the full adjusted multivariable model and should not be interpreted as the square of the Spearman correlation reported in Table 4.

A sensitivity analysis of linearity indicated a modest departure from strict linearity. Adding a quadratic PES-NWI term increased the explained variance from R^2^ = 0.230 to R^2^ = 0.246 (ΔR^2^ = 0.016), representing a 1.6-percentage-point improvement in explained variance. The quadratic term was statistically significant using HC3-robust standard errors (*p* = 0.019). Thus, although the overall association remained positive, the linear coefficient should be interpreted as an average association across the observed PES-NWI range rather than as a constant effect at every score level.

Aside from the modest departure from strict linearity described above, the remaining regression diagnostics indicated no major violations of model assumptions. Standardized residuals were approximately normally distributed (Shapiro–Wilk *p* = 0.075; skewness = −0.144; kurtosis = −0.387), and the residual-versus-predicted plot showed no marked systematic pattern. Standardized residuals ranged from −2.81 to 2.17 and studentized residuals from −2.83 to 2.20, with no observations exceeding an absolute value of 3. The maximum Cook’s distance was 0.03. One observation had a relatively elevated leverage value of 0.13; however, its studentized residual was 0.56 and Cook’s distance was negligible, indicating that it was not influential.

A separate complete-case sensitivity analysis restricted to participants with valid responses to all 14 CCB items (*n* = 345) yielded substantively similar results. The PES-NWI composite remained positively correlated with CCB (ρ = 0.330, *p* < 0.001), and the adjusted association remained statistically significant (B = 1.106, HC3-robust standard error = 0.188, 95% confidence interval = 0.737–1.475, *p* < 0.001). The complete-case adjusted model explained 26.9% of the variance in CCB (R^2^ = 0.269; adjusted R^2^ = 0.238), and adding PES-NWI to the covariate-only model increased explained variance by 10.1 percentage points (ΔR^2^ = 0.101). These findings indicate that the primary results were robust to the CCB item-completeness criterion (Supplementary Table S1).

### 3.6. Comparisons of Culturally Competent Behaviors Across Participant Characteristics

CCB scores differed significantly by age group (H(2) = 8.98, *p* = 0.011), gender (U = 4928.00, *p* < 0.001), nationality (H(3) = 11.12, *p* = 0.011), and years of nursing experience (H(3) = 17.10, *p* < 0.001). No statistically significant differences were observed by study site (H(2) = 3.95, *p* = 0.139), religion (U = 16,316.50, *p* = 0.601), or educational level (U = 11,059.50, *p* = 0.792) (Table 6).

## 4. Discussion

This study found that a more favorable nursing practice environment was associated with more frequent culturally competent behaviors among surgical nurses in Riyadh. The association remained statistically significant after adjustment for study site and selected demographic and professional factors. The complete-case sensitivity analysis yielded substantively similar bivariate and adjusted estimates, indicating that the main conclusion was not dependent on the study-specific CCB item-completeness criterion. Although the adjusted model explained 23.0% of the variance in culturally competent behavior scores, a substantial proportion of the variance remained unexplained, indicating that other individual, organizational, and contextual factors not included in the present model may also contribute to culturally competent behaviors.

Cultural competence is often viewed as an individual attribute involving knowledge, awareness, attitudes, and behavior [1,9]. However, nurses work within organizational systems that may support or constrain its application in practice. The Cultural Congruence Model conceptualizes culturally congruent care as an interaction among the professional, the patient, and the healthcare environment [16], whereas Donabedian’s framework suggests that organizational structures influence care processes [19]. Nurses may therefore understand cultural needs but struggle to respond when staffing, communication resources, or managerial support is limited. This is relevant because the CCB subscale assesses culturally competent behavior rather than knowledge alone [17,18]. Because of the cross-sectional design, the direction of the observed association cannot be established. Although the conceptual framework proposes that a supportive nursing practice environment may facilitate culturally competent behaviors, the reverse direction is also plausible. Nurses who more frequently engage in culturally competent behaviors may develop more effective relationships with patients and colleagues, which could, in turn, contribute to more favorable perceptions of the practice environment, particularly collegial nurse–physician relations. The observed association should therefore be interpreted as potentially bidirectional, and longitudinal studies are needed to clarify temporal direction.

The findings are consistent with earlier research linking workplace conditions with culturally responsive care. Teixeira et al. reported associations among cultural competence, the nursing work environment, and culturally congruent care in multicultural units [15]. Wesołowska et al. also reported an association between the psychosocial work environment and cross-cultural competence among nurses [20]. Lee and Choi examined individual and organizational factors related to maternal-newborn nurses’ cultural competency [21], whereas Schenk et al. examined organizational and staff-related factors associated with cultural competence among hospital staff [22]. However, these studies were conducted in healthcare systems and cultural contexts that differ from those in Saudi Arabia; therefore, direct comparisons should be made cautiously. Taken together, these studies suggest that culturally competent behavior may be partly related to the conditions in which nurses practice.

This relationship is especially relevant in Saudi Arabia, where a culturally diverse nursing workforce cares for patients with varied linguistic, cultural, and religious needs [2,5]. Language barriers, cultural differences, and limited preparation for the local context have been reported [2,6]. Falatah et al. linked cultural competence with structural empowerment and effective communication [23]. The Saudi literature has highlighted education and training in cultural competence [24,25,26] and the need for care that reflects patients’ cultural and religious expectations [27]. Chae and Park also emphasized the role of organizational cultural competence in supporting care for diverse patients [28].

The secondary domain-level analyses provide additional context for the overall association. All five PES-NWI domains were positively associated with CCB, with the largest coefficient observed for nurse participation in hospital affairs (ρ = 0.332), followed by nursing foundations for quality of care (ρ = 0.309); the remaining domain coefficients ranged from 0.249 to 0.275. Because these analyses were unadjusted and the domains represent related dimensions of the same practice environment, differences in the correlation coefficients should not be interpreted as independent domain effects or as a causal ranking of organizational priorities. Nevertheless, the consistently positive pattern across all five domains suggests that the observed relationship is not confined to a single aspect of the practice environment. The comparatively larger association for nurse participation may be hypothesis-generating, potentially reflecting the importance of professional involvement and organizational voice in nurses’ ability to adapt care to diverse patient needs.

Staffing and resource adequacy warrants separate attention because it was the least favorably rated PES-NWI domain, even though its bivariate association with CCB was not the strongest. Culturally competent care may require additional time and resources for communication, assessment of patient preferences, education, family involvement, and interpreter use. Supportive work environments have been associated with better nurse and patient outcomes [29], reduced adverse effects of burnout on patient outcomes [30], and improved job-related outcomes among nurses in Saudi Arabia [31]. Although these outcomes were not measured in the present study, limited staffing and resources may constrain nurses’ ability to provide individualized, culturally responsive care consistently.

This may be particularly important in surgical settings, where communication about preparation, pain management, postoperative care, and discharge is often time-sensitive. Language and cultural differences may affect patients’ understanding and participation in decisions [6,7], while beliefs about pain, modesty, family roles, and gender preferences may shape care expectations [4,8,27]. Surgical nurses therefore need both personal competence and organizational support.

The moderate unadjusted correlation and the 9.1-percentage-point increase in explained variance after adding PES-NWI to the covariate-only model suggest that the practice environment is a meaningful but partial correlate of culturally competent behavior. Other factors reported in the literature include educational level, cultural diversity training, empathy, nurse-to-patient ratios, prior work experience, and organizational feedback systems [32,33,34]. Healthcare organizations should therefore avoid treating practice-environment improvement as a stand-alone strategy and instead combine staff development with organizational approaches such as language support, culturally adapted information, and policies that facilitate culturally responsive care [35,36]. Although educational interventions can improve cultural competence [37,38,39], such efforts are likely to be more effective when supported by organizational resources and leadership [35,40].

### Limitations

This study has several limitations. Its cross-sectional design prevents conclusions about causality or the direction of the observed association. The nursing practice environment and culturally competent behaviors were assessed using self-report questionnaires, which may be subject to social desirability and common-method bias. The study included only surgical nurses working in participating healthcare institutions in Riyadh, limiting the generalizability of the findings to other specialties, regions, and healthcare systems. The use of convenience sampling may have introduced selection bias and limited the representativeness and external validity of the findings. Although all eligible surgical nurses at the participating institutions were invited, the response rate was 41.0%; therefore, non-response bias cannot be excluded. CCB scores could not be calculated for 18 participants (4.3%) because “not sure” responses were not scored and fewer than 11 valid CCB items remained under the study-specific completeness criterion. Although 404 participants (95.7%) were retained in the CCB analyses, participants with and without calculable CCB scores differed by study site and nationality; these differences should therefore be considered when interpreting the findings. In addition, the model did not include potentially relevant factors, including language proficiency, cultural competence training, cultural exposure, workload, interpreter availability, and organizational cultural competence. Future research should directly assess these structural factors, particularly workload and interpreter availability, to determine whether they are independently associated with culturally competent behaviors beyond the dimensions captured by the PES-NWI. The dichotomous categorization of religion as Muslim and Others may have obscured heterogeneity among non-Muslim participants. Potential interactions between study site and participant nationality or religion were not examined because these analyses were not prespecified, and the study was not specifically powered to detect interaction effects. Future studies with larger samples should examine whether the association between the nursing practice environment and culturally competent behaviors varies across institutional and cultural subgroups.

## 5. Conclusions

The nursing practice environment had a moderate positive unadjusted association with culturally competent behavior scores, and this association remained statistically significant after adjustment for study site and selected demographic and professional characteristics. Although the practice environment was rated favorably overall, staffing and resource adequacy received the lowest domain score, suggesting an area that may require organizational attention. Healthcare institutions may consider strengthening staffing and resource support, nursing leadership, nurses’ participation in decision-making, interprofessional collaboration, and access to culturally appropriate communication and educational resources. Because causal conclusions cannot be drawn, longitudinal and intervention studies are needed to determine whether improvements in the nursing practice environment lead to more culturally competent care and better patient outcomes.

## Figures and Tables

**Table 1:** Participants’ demographic and professional characteristics (N = 422).

Characteristics	*n* (%)
**Gender**	
Female	377 (89.3)
Male	45 (10.7)
**Age group**	
26–35 years	173 (41.0)
36–45 years	160 (37.9)
≤25 or ≥46 years	89 (21.1)
**Study site**	
Site A	146 (34.6)
Site B	139 (32.9)
Site C	137 (32.5)
**Nationality**	
Indian	168 (39.8)
Filipino	124 (29.4)
Saudi	70 (16.6)
Other nationalities	60 (14.2)
**Religion**	
Muslim	123 (29.1)
Others	299 (70.9)
**Education**	
Bachelor’s degree or higher	354 (83.9)
Diploma	68 (16.1)
**Nursing experience**	
1–3 years	78 (18.5)
4–6 years	67 (15.9)
7–9 years	75 (17.8)
≥10 years	202 (47.9)

**Table 2:** Internal consistency reliability of study measures.

Measure	Number of Items	Cronbach’s Alpha
Practice Environment Scale of the Nursing Work Index (PES-NWI)	31	0.964
Nurse participation in hospital affairs	9	0.926
Nursing foundations for quality of care	10	0.881
Nurse-manager ability, leadership and support of nurses	5	0.818
Staffing and resource adequacy	4	0.838
Collegial nurse–physician relations	3	0.802
Cultural Competence Behaviors (CCB)	14	0.955

The CCB reliability analysis used complete cases across all 14 items (N = 345).

**Table 3:** Descriptive statistics of the nursing practice environment and culturally competent behaviors among surgical nurses.

Measure	N	Mean	SD
Cultural Competence Behaviors (CCB)	404	4.48	1.53
Nursing Practice Environment (PES-NWI)	422	3.04	0.46
Nurse participation in hospital affairs	422	3.03	0.54
Nursing foundations for quality of care	422	3.16	0.43
Nurse-manager ability, leadership and support of nurses	422	3.04	0.51
Staffing and resource adequacy	422	2.87	0.63
Collegial nurse–physician relations	422	3.11	0.50

Abbreviations: CCB, Cultural Competence Behaviors; PES-NWI, Practice Environment Scale of the Nursing Work Index; SD, standard deviation.

**Table 4:** Associations of the PES-NWI composite score and domains with culturally competent behaviors (N = 404).

Association	Spearman’s ρ	*p*-Value
PES-NWI composite and culturally competent behaviors	0.303	<0.001
Nurse participation in hospital affairs	0.332	<0.001
Nursing foundations for quality of care	0.309	<0.001
Nurse-manager ability, leadership and support of nurses	0.265	<0.001
Staffing and resource adequacy	0.275	<0.001
Collegial nurse–physician relations	0.249	<0.001

Abbreviation: PES-NWI, Practice Environment Scale of the Nursing Work Index.

**Table 5:** Adjusted association between the nursing practice environment and culturally competent behaviors.

Primary Predictor	B	HC3-Robust SE	95% CI	*p*-Value
Nursing Practice Environment (PES-NWI)	1.037	0.172	0.699–1.375	<0.001

**Table 6:** Culturally competent behavior scores across demographic and professional characteristics (N = 404).

Characteristic/Group	*n*	Median (Q1–Q3)	Test Statistic	*p*-Value
**Age group**			H(2) = 8.978	0.011
26–35 years	167	4.43 (3.21–5.42)		
36–45 years	150	4.43 (3.55–5.86)		
≤25 or ≥46 years	87	4.86 (4.07–6.07)		
**Gender**			U = 4928.00	<0.001
Female	360	4.61 (3.57–5.85)		
Male	44	3.63 (2.00–4.32)		
**Study site**			H(2) = 3.947	0.139
Site A	144	4.52 (3.79–5.86)		
Site B	123	4.50 (3.50–5.57)		
Site C	137	4.43 (2.64–5.73)		
**Nationality**			H(3) = 11.118	0.011
Indian	156	4.27 (3.25–5.63)		
Filipino	123	4.93 (3.62–6.21)		
Saudi	68	4.36 (3.26–5.57)		
Other nationalities	57	4.36 (3.84–5.07)		
**Religion**			U = 16,316.50	0.601
Muslim	118	4.43 (3.63–5.57)		
Others	286	4.52 (3.43–5.86)		
**Educational level**			U = 11,059.50	0.792
Diploma	67	4.64 (3.25–5.71)		
Bachelor’s degree or higher	337	4.50 (3.54–5.71)		
**Years of nursing experience**			H(3) = 17.101	<0.001
1–3 years	73	4.29 (2.33–5.16)		
4–6 years	65	4.27 (3.36–5.71)		
7–9 years	73	4.43 (3.32–5.54)		
≥10 years	193	4.67 (3.86–5.96)		

Values are presented as median (Q1–Q3). Mann–Whitney U tests were used for two-group comparisons and Kruskal–Wallis H tests for comparisons involving three or more groups. Q1 and Q3 denote the 25th and 75th percentiles, respectively.

## Data Availability

The data supporting the findings of this study are available from the corresponding author upon reasonable request. The data are not publicly available because of ethical and privacy considerations. The same underlying dataset was also used in a separate manuscript that addresses a distinct research question and objective and employs a different statistical analysis.

## Supplementary Materials

The following supporting information can be downloaded at https://www.mdpi.com/article/10.3390/healthcare14183017/s1, Table S1. Sensitivity analysis comparing the primary CCB scoring sample with the complete-case sample.

## Abbreviations

The following abbreviations are used in this manuscript:

CCA	Cultural Competence Assessment
CCB	Cultural Competence Behavior
CI	Confidence Interval
HC3	Heteroscedasticity-consistent type 3
IRB	Institutional Review Board
PES-NWI	Practice Environment Scale of the Nursing Work Index
SPSS	Statistical Package for the Social Sciences
VIF	Variance inflation factor
