# Supplementary material for: The Association Between the Nursing Practice Environment and Culturally Competent Behaviors Among Surgical Nurses in Riyadh, Saudi Arabia: A Multicenter Cross-Sectional Study
Source: Healthcare (Basel). 2026 Sep 15;14(18):3017. doi: 10.3390/healthcare14183017 (PMC13606369; doi:10.3390/healthcare14183017)
Supplement: Supplementary file 1 [file healthcare-14-03017-s001.zip › healthcare-4526212-supplementary.pdf]

# Supplementary Material

## Supplementary Table S1. Sensitivity analysis comparing the primary CCB scoring sample with the complete-case sample

| Statistic / analysis | Primary sample ≥11/14 valid CCB items (n = 404) | Complete-case sample 14/14 valid CCB items (n = 345) |
|---|---|---|
| **A. Primary outcome and adjusted model** | | |
| CCB mean ± SD | 4.484 ± 1.527 | 4.506 ± 1.556 |
| Spearman's ρ: PES-NWI composite vs. CCB | 0.303 ($p < 0.001$) | 0.330 ($p < 0.001$) |
| Adjusted PES-NWI coefficient, B | 1.037 | 1.106 |
| HC3 robust SE | 0.172 | 0.188 |
| 95% CI for B | 0.699–1.375 | 0.737–1.475 |
| p value for adjusted PES-NWI coefficient | <0.001 | <0.001 |
| Full-model $R^2$ | 0.230 | 0.269 |
| Adjusted $R^2$ | 0.202 | 0.238 |
| Covariate-only $R^2$ | 0.139 | 0.167 |
| $\Delta R^2$ after adding PES-NWI | 0.091 | 0.101 |
| **B. Domain-level Spearman correlations with CCB** | | |
| Nurse participation in hospital affairs | 0.332 ($p < 0.001$) | 0.357 ($p < 0.001$) |
| Nursing foundations for quality of care | 0.309 ($p < 0.001$) | 0.347 ($p < 0.001$) |
| Nurse-manager ability, leadership and support of nurses | 0.265 ($p < 0.001$) | 0.297 ($p < 0.001$) |
| Staffing and resource adequacy | 0.275 ($p < 0.001$) | 0.302 ($p < 0.001$) |
| Collegial nurse–physician relations | 0.249 ($p < 0.001$) | 0.278 ($p < 0.001$) |
| **C. Linearity sensitivity** | | |
| $\Delta R^2$ after adding quadratic PES-NWI term | 0.016 | 0.018 |
| HC3 p value for quadratic term | 0.019 | 0.024 |

**Note.** CCB = Cultural Competence Behaviors; PES-NWI = Practice Environment Scale of the Nursing Work Index; HC3 = heteroscedasticity-consistent type 3; SE = standard error; CI = confidence interval. The primary CCB score was calculated when at least 11 of 14 CCB items had valid scored responses; the complete-case sensitivity analysis was restricted to participants with valid responses to all 14 CCB items. Both adjusted models included study site, age group, gender, nationality, religion, educational level, and nursing experience as covariates. In Panel A, $\Delta R^2$ denotes the increase in explained variance after adding the PES-NWI composite score to the covariate-only model. In Panel C, $\Delta R^2$ denotes the increase after adding a quadratic PES-NWI term to the adjusted linear model. All tests were two-sided.
